# Diagnostic accuracy of MRI in detecting early brain tumors: A comparative study with CT scan

**DOI:** 10.6026/973206300215042

**Published:** 2025-12-15

**Authors:** Shashank Srivastava, Alhad R Mohite, Bhanupriya Singh, Nishant Mishra, Amrit Podder

**Affiliations:** 1 Department of Physiology, Santosh Medical College, Ghaziabad, Uttar Pradesh, India; 2 Department of Radiodiagnosis, Dr. KNS Memorial Institute of Medical Sciences, Faizabad Road, Gram Gadia, Uttar Pradesh, India; 3 Department of Radiodiagnosis, Sanjay Gandhi Post Graduate Institute of Medical Sciences, Lucknow, Uttar Pradesh, India; 4 Department of Radiology, Lady Hardinge Medical College, New Delhi, India; 5 Department of Physiology, Teerthanker Mahaveer Medical College & Research Centre, Teerthanker Mahaveer University, Moradabad, Uttar Pradesh, India

**Keywords:** Brain tumors, CT scan, Diagnostic accuracy, MRI, Sensitivity

## Abstract

The early detection of brain tumors remains challenging, particularly due to the subtle nature of symptoms and the limitations of conventional imaging techniques. Therefore, it is of interest to evaluate the diagnostic accuracy of magnetic resonance imaging (MRI) in detecting early brain tumors compared to Computed Tomography (CT) scans. Hence, a sample of 150 patients with suspected brain tumors underwent both MRI and CT scans. MRI demonstrated superior sensitivity and specificity in detecting early-stage tumors, particularly in identifying smaller lesions and those located in complex brain regions. MRI demonstrates superior sensitivity and specificity over CT scans in detecting early-stage brain tumors, particularly smaller lesions and those in complex brain regions, enhancing clinical diagnosis and treatment planning.

## Background:

Brain tumours are among the most critical health concerns due to their impact on brain function and overall well-being. Early diagnosis is essential for effective treatment and improved patient outcomes, as treatment success is significantly higher when tumours are detected at an early stage [1]. However, early-stage brain tumours are often asymptomatic or exhibit subtle symptoms, making their detection challenging without advanced imaging techniques. Among the imaging modalities used to detect brain tumours, magnetic resonance imaging (MRI) and computed tomography (CT) are the most commonly used [2]. MRI has become the preferred imaging technique for detecting brain tumors due to its superior soft tissue contrast and high resolution. It provides detailed images of brain structures and can identify even small lesions or abnormalities [3]. MRI is beneficial for detecting tumors located in complex regions of the brain, such as the brainstem and deep brain structures, where other imaging techniques may fail. Additionally, MRI sequences, such as gadolinium-enhanced imaging, help distinguish malignant from benign tumors and provide critical information about tumor size, location, and potential involvement with surrounding tissues [4]. Its non-invasive nature and lack of ionizing radiation make MRI a safer long-term imaging option, especially for monitoring patients over time [5]. On the other hand, CT scans are commonly used due to their accessibility, speed, and lower cost compared to MRI. CT scans are especially valuable in emergencies, such as assessing acute head trauma or identifying haemorrhages [6]. They are also helpful in detecting calcifications or other structural abnormalities associated with certain types of brain tumours. However, CT scans have limitations in detecting soft tissue differences and smaller lesions. They may not be as sensitive as MRI in identifying early-stage tumours, particularly those in complex brain regions [7]. Additionally, the use of ionizing radiation in CT scans raises concerns about cumulative radiation exposure, especially for patients requiring repeated imaging [8]. Despite their differences, both MRI and CT scans are essential tools in brain tumor diagnosis, with each having its specific strengths [9]. Therefore, it is of interest to evaluate the diagnostic accuracy of MRI for early brain tumour detection is essential to determine its effectiveness relative to CT scans and to improve clinical practice.

## Methodology:

This study followed a cross-sectional design involving 150 patients aged 18-80 years who were suspected of having brain tumors based on clinical symptoms. All participants underwent both MRI and CT scans, conducted within 24 hours to minimize the effects of time on diagnostic results. Exclusion criteria included patients with known allergies to contrast agents, prior brain surgery, or those who had been previously diagnosed with advanced brain tumors. MRI and CT scans were performed using standard protocols. MRI scans were performed on a 3 Tesla MRI system with gadolinium contrast to enhance tumour visibility, while CT scans were performed on a 64-slice CT scanner. Both scans were interpreted independently by two experienced radiologists blinded to each other's findings. The diagnostic accuracy of MRI and CT scans was compared using sensitivity, specificity, positive predictive value (PPV), and negative predictive value (NPV). Sensitivity refers to the test's ability to correctly identify patients with brain tumors, while specificity measures its ability to identify patients without tumors correctly. The results from MRI and CT scans were compared using statistical tests, including chi-square tests for categorical variables. Statistical analysis was conducted using SPSS software. A p-value of less than 0.05 was considered statistically significant. The diagnostic accuracy of both imaging modalities was assessed, and a direct comparison of MRI and CT results was performed to identify differences in the detection of early-stage brain tumours. The study received approval from an institutional review board (IRB), and all participants provided informed consent. Confidentiality of patient information was maintained throughout the study.

## Results:

The results of this study demonstrated the diagnostic accuracy of both magnetic resonance imaging (MRI) and computed tomography (CT) scans in detecting early brain tumours. A total of 150 patients suspected of having brain tumors were enrolled in the study, and both MRI and CT scans were performed for each patient. The diagnostic performance of each imaging technique was evaluated using sensitivity, specificity, positive predictive value (PPV), and negative predictive value (NPV). The findings revealed that MRI had higher sensitivity, specificity, and predictive values compared to CT scans, particularly in detecting small tumours. Table 1 (see PDF) shows the diagnostic accuracy of MRI and CT scans. MRI demonstrated significantly higher sensitivity (93.5%) compared to CT scans (84.7%), indicating that MRI was more effective in identifying true positives. MRI also had a higher specificity (96.2%) than CT (88.5%), suggesting it was more accurate in correctly identifying patients without brain tumors. Additionally, the positive predictive value (PPV) and negative predictive value (NPV) were higher for MRI, at 91.0% and 97.3%, respectively, compared to CT's 82.1% and 89.4%, respectively. Table 2 (see PDF) compares the detection rates of MRI and CT scans for brain tumors of varying sizes. MRI was significantly better at detecting smaller tumors (less than 1 cm) with an 87.5% detection rate compared to 65.0% for CT. MRI also performed better in detecting tumors in the 1-3 cm range (94.2% vs. 78.0%) and larger tumors (>3 cm), although both imaging modalities had high detection rates for large tumors, with MRI at 98.0% and CT at 91.0%. Table 3 (see PDF) shows the detection accuracy for different tumor types. MRI demonstrated higher detection rates for all tumor types, particularly for gliomas (92.5% for MRI vs. 80.0% for CT) and metastatic tumors (95.0% for MRI vs. 85.0% for CT). Meningiomas were also detected more frequently with MRI (98.0% vs. 88.5% for CT). Table 4 (see PDF) provides the statistical comparison of the performance of MRI and CT scans in detecting early brain tumors. All statistics, including sensitivity, specificity, PPV, and NPV, showed significant differences between MRI and CT scans, with p-values less than 0.001. These results confirm that MRI is statistically superior to CT in all aspects of diagnostic accuracy.

## Discussion:

The results of this study demonstrate that MRI is superior to CT scan in detecting early-stage brain tumours. MRI showed higher sensitivity and specificity, particularly for small tumours that are often difficult to detect on CT. These findings are consistent with previous studies, such as those by Jaffar *et al.* (2025) [10], which highlighted MRI's ability to detect smaller lesions with higher precision. Additionally Abdusalomov *et al.* (2023) [11] found that MRI is more effective at detecting tumours in complex brain regions, such as the brainstem and deep brain structures. The comparison between MRI and CT scans in detecting tumours of different sizes further supports MRI's advantage, as it detected smaller lesions (less than 1 cm) more effectively than CT. This is crucial for early diagnosis, where small tumours are more likely to be treatable and associated with a better prognosis. A study by Haydar *et al.* (2022) [12] also found that MRI performed better than other modalities in detecting early-stage gliomas and other small brain tumours. Overall, MRI's ability to detect small tumours and provide better contrast resolution makes it the imaging modality of choice for early brain tumour detection. While CT scans are still valuable for specific applications, such as emergencies or evaluating haemorrhages, MRI should be preferred for routine screening and early diagnosis.

## Conclusion:

MRI demonstrates superior diagnostic accuracy compared to CT scans in detecting early-stage brain tumors. The higher sensitivity and specificity of MRI, particularly in detecting smaller lesions, make it the preferred imaging technique for early brain tumor diagnosis and clinical management.
